# Multidimensional Frailty-Related Vulnerability Profiles Among Community-Dwelling Older Adults: A Data-Driven Cluster Analysis of the 2023 National Survey of Older Koreans

**DOI:** 10.3390/healthcare14213585

**Published:** 2026-10-23

**Authors:** Muncheong Choi, Alchan Kim

**Affiliations:** 1 Department of Kinesiology and Sports Studies, College of Science and Industry Convergence, Ewha Womans University, Seoul 03760, Republic of Korea; muncheong.choi@gmail.com; 2 Department of Sports & Technology, Seokyeong University, 124 Seogyeong-ro, Seongbuk-gu, Seoul 02713, Republic of Korea

**Keywords:** frailty, health behavior, walking, cognition, community-dwelling, depressive symptoms

## Abstract

**Highlights:**

**What are the main findings?**

- We used K-means clustering to identify a lower-vulnerability profile and a multidimensional higher-vulnerability profile among 9950 community-dwelling older adults.
- Among the indicators used to derive the clusters, mobility-related measures showed the largest descriptive separation. This finding does not establish their independent clinical importance.

**What are the implications of the main findings?**

- The derived profiles describe how functional, health-related, cognitive, and psychological vulnerabilities co-occur within the analytical sample. Whether these profiles provide information beyond established frailty screening tools requires further validation.
- Independent and longitudinal validation is needed to determine whether these profiles have additional clinical utility for assessment or preventive planning.

**Abstract:**

**Background/Objectives:** Frailty-related vulnerability spans health, physical function, health behaviors, cognition, and psychological well-being. A single summary score may not fully convey how these features co-occur within individuals. This study identified data-driven frailty-related profiles among community-dwelling older adults and compared the magnitude of between-profile differences. **Methods:** We conducted a cross-sectional secondary analysis of the 2023 National Survey of Older Koreans. The analytical sample included 9950 participants. Ten indicators—self-rated health, chronic disease count, 1-year weight loss, sleep quality, weekly exercise frequency, fatigue, stair-climbing ability, 300-m walking ability, cognitive function, and depressive symptoms—were standardized and entered into K-means clustering. Candidate solutions for K = 2–10 were evaluated using within-cluster sums of squares and mean silhouette coefficients. Profile characteristics and effect sizes were compared using independent-samples *t*-tests, Pearson’s chi-squared tests, Cohen’s *d*, and Cramér’s *V*. **Results:** The K = 2 solution had the highest mean silhouette coefficient (0.273) among the candidate solutions, indicating modest separation between clusters. Within the analytical sample, 6962 participants (70.0%) formed a lower-vulnerability profile and 2988 (30.0%) a multidimensional higher-vulnerability profile. The latter profile showed less favorable health, functional, psychological, and cognitive characteristics. Among the cluster-defining indicators, the largest descriptive differences were observed in 300-m walking (*d* = 2.013) and stair-climbing ability (*d* = 2.001). **Conclusions:** The two clusters represented a relative multidimensional vulnerability gradient rather than clearly distinct clinical phenotypes. Although the findings describe co-occurring vulnerability characteristics, their potential clinical utility requires independent and longitudinal validation.

## 1. Introduction

Frailty is a state of heightened vulnerability in later life that arises as physiological reserve and resilience decline across multiple systems. It has been associated with disability, falls, hospitalization, institutionalization, and mortality, making its recognition relevant to healthcare planning for aging populations [1,2,3,4]. Rather than representing a single disease, frailty captures susceptibility, which often intersects with chronic illnesses, functional limitations, and poor psychosocial health. Therefore, earlier identification of this broader vulnerability may support preventive assessments before adverse outcomes are established.

Several complementary frameworks have been developed to assess frailty, each capturing vulnerability from a different perspective. Fried et al. defined a physical phenotype based on unintentional weight loss, exhaustion, weakness, slow walking speed, and low physical activity [2], whereas the deficit-accumulation approach quantifies the proportion of health deficits across a broad range of variables [3]. Brief instruments such as the FRAIL scale, including its validated Korean version (K-FRAIL), further translate core frailty domains into clinically feasible screening questions [4,5]. These approaches remain valuable for summarizing overall vulnerability; however, their reliance on aggregate scores or predefined categories inevitably compresses information about how functional, health-related, behavioral, cognitive, and psychological vulnerabilities coexist within individuals. Therefore, examining these domains jointly at the individual level is important for identifying clinically meaningful patterns of multidimensional vulnerability that conventional summary measures may obscure.

Person-centered data-driven methods provide a direct way to examine how multiple frailty-related characteristics cluster within individuals rather than reducing them to a single summary score. Cluster analysis, latent class analysis, and latent profile analysis jointly evaluate patterns across health, physical function, health behavior, cognition, and psychological status and group individuals with similar multivariable profiles. Because these groups are derived from observed data rather than predefined clinical cut points, they can identify combinations of vulnerability that conventional summary measures may conceal. Their value therefore lies in characterizing the structure of multidimensional vulnerability across clinically relevant domains, while the resulting profiles remain descriptive rather than diagnostic.

International studies have illustrated a range of patterns that are detectable using person-centered analyses. In a large secondary analysis of older adults, Looman et al. used latent class analysis across self-reported health, cognition, social functioning, mental health, morbidity, and functional limitations to identify six frailty profiles [6]. Bohn et al. combined factor analysis with latent profile analysis to characterize early frailty patterns among community-dwelling adults and found three profiles that differed in multidomain vulnerability [7]. Although their measures and analytical methods differ, these studies indicate that frailty-related vulnerabilities do not necessarily appear in a single uniform configuration.

Evidence from Korea has also begun to examine multidimensional frailty using person-centered methods. Han, Kim, and Cho analyzed 3824 men aged ≥65 years from the 2023 National Survey of Older Koreans using latent class analysis, identified four multidimensional frailty profiles, and examined their associations with life satisfaction [8]. This study establishes the relevance of data-driven profiling in the Korean context while leaving room to evaluate broader analytical samples that include both women and men and integrate health, physical function, health behavior, cognitive, and psychological indicators. Evidence remains limited regarding the magnitude of between-profile differences across the indicators that underlie such profiles.

Accordingly, this study aimed to identify data-driven frailty-related profiles among community-dwelling older adults using health, physical function, health behavior, and cognitive and psychological indicators from the 2023 National Survey of Older Koreans. The present study was designed to characterize how multiple frailty-related dimensions align within a large sample of older women and men, rather than to establish new clinical frailty categories. The descriptive magnitude of differences across cluster-defining indicators, together with sensitivity to alternative analytical specifications, provides information on the structure and methodological dependence of the observed vulnerability partition.

## 2. Materials and Methods

### 2.1. Study Design and Participants

This study was a cross-sectional secondary analysis of data from the 2023 National Survey of Older Koreans (NSOK). The NSOK was a nationwide survey of community-dwelling adults aged ≥65 years across 17 provinces and metropolitan cities in the Republic of Korea and used a stratified cluster sampling design. The survey collected comprehensive information on health and functional, psychological, and social characteristics. Of the 10,078 survey respondents, 128 were excluded from the primary analytical sample during data preparation, leaving 9950 participants for analysis. To assess potential selection bias associated with these exclusions, available demographic and functional characteristics were compared between included and excluded respondents.

### 2.2. Selection and Definition of Variables

We selected the clustering variables to anchor the analysis in the core frailty domains represented by K-FRAIL while extending the model to capture broader multidimensional vulnerability available in the 2023 NSOK. Rather than applying K-FRAIL as a diagnostic instrument, we used its five core domains to guide variable selection: fatigue, ability to climb 10 stairs without assistance, ability to walk 300 m without assistance, chronic disease count, and 1-year weight change (kg) were mapped to fatigue, resistance, ambulation, illness, and loss of weight, respectively. We then incorporated self-rated health, sleep quality, weekly exercise frequency, cognitive function, and depressive symptoms because these indicators capture clinically relevant dimensions of health, health behavior, cognition, and psychological well-being that are not fully represented by the five K-FRAIL domains alone. This approach yielded a 10-variable set designed to characterize multidimensional frailty-related vulnerability more comprehensively within the 2023 NSOK dataset. The variables used for clustering and profile characterization, together with their roles in the analysis, are summarized in Table 1.

#### 2.2.1. Variables Used for Cluster Analysis

The fatigue item (C3) ranged from 1 (always tired) to 5 (never tired), with higher scores indicating less frequent fatigue. The stair-climbing (C4) and 300-m walking (C5) items retained their original coding of 1 (difficulty) and 2 (no difficulty); thus, higher scores indicated better mobility. The chronic disease count (CL_B3C) was the total number of physician-diagnosed chronic diseases (range: 0–15). The continuous 1-year weight-change indicator was calculated by subtracting current body weight (B7_1) from body weight 1 year previously (B7_3), expressed in kilograms. Positive values indicated weight loss, negative values indicated weight gain, and zero indicated no change. The indicator did not distinguish intentional from unintentional weight loss.

Self-rated health (B1) ranged from 1 (very healthy) to 5 (not healthy at all), and sleep quality (B2) ranged from 1 (slept very well) to 5 (did not sleep well at all); higher values on both measures indicated a worse status. Weekly exercise frequency (days/week) was coded as 0 days/week for participants reporting no regular exercise and as the reported frequency of 1–7 days/week among regular exercisers. Korean Mini-Mental State Examination-2 (K-MMSE-2) scores ranged from 0 to 30, with higher scores indicating better cognitive function. The 15-item Short Geriatric Depression Scale (SGDS-15) scores ranged from 0 to 15, with higher scores indicating more severe depressive symptoms. All 10 clustering variables were standardized as Z-scores before K-means clustering.

#### 2.2.2. Variables Used for Profile Characterization

The derived profiles were characterized using age, sex, educational level, living arrangement, activities of daily living (ADL), fatigue status, categorized self-rated health, poor sleep, and cognitive impairment status. Educational attainment was grouped into five categories: no formal education, elementary school, middle school, high school, and college or higher, while living alone was coded as a binary variable. Basic functional independence was quantified by summing seven ADL items scored as 1 (fully independent), 2 (partial assistance), or 3 (complete assistance), producing a total score ranging from 7 to 21; higher scores indicated greater dependence in daily activities.

Self-rated health was categorized as good (B1 = 1–2), fair (B1 = 3), or poor (B1 = 4–5). Poor sleep was operationally defined as a response of 4 or 5 on B2. Fatigue was defined according to the K-FRAIL criteria as feeling tired always or most of the time on C3. Cognitive impairment was defined using the TQIK2_1.5sd variable provided in the 2023 NSOK, which applies an age- and education-adjusted 1.5-standard-deviation criterion to the K-MMSE-2 score. Because the cognitive impairment indicator was derived from K-MMSE-2, which was included in the clustering model, its between-cluster comparison was used solely for descriptive characterization and was not considered independent validation. The distributions of these clustering-related descriptive characteristics were reported separately from characteristics not used for clustering (Table S1).

### 2.3. Statistical Analyses

Standardization of the clustering variables, estimation of K-means solutions for K = 2–10, and calculation of the within-cluster sum of squares (WCSS) and mean silhouette coefficients were performed using Python 3.13 (Python Software Foundation, Beaverton, OR, USA) and scikit-learn 1.8.0 (scikit-learn developers, open-source project). Descriptive statistics, between-profile comparisons, and effect size calculations were performed using IBM SPSS Statistics for Windows (version 25.0; IBM Corp., Armonk, NY, USA). Additional stability and sensitivity analyses were conducted to examine the dependence of the clustering results on initialization, sampling variation, measurement characteristics, indicator selection, and survey weighting.

The ten clustering variables were standardized as Z-scores using StandardScaler. K-means clustering was performed using Euclidean distance, k-means++ initialization, n_init = 10, and random_state = 42. Candidate solutions from K = 2 to K = 10 were evaluated using WCSS and mean silhouette coefficients. WCSS was obtained from the inertia value of the scikit-learn K-means estimator, and silhouette coefficients were calculated using Euclidean distance. The number of clusters was selected by considering the silhouette coefficient, WCSS pattern, parsimony, and substantive interpretability together. For the selected solution, the proportion of total standardized variation attributable to between-cluster separation was summarized as BSS/TSS = (TSS − WCSS)/TSS. K-means was used as an exploratory, centroid-based approach to characterize patterns in standardized multivariable space. Although Z-score standardization reduced differences in numerical scale, it did not eliminate differences in measurement properties among continuous, ordinal, and binary indicators. The resulting clusters were therefore interpreted as an empirical partition conditional on the specified distance measure and indicator set.

Two procedures were used to evaluate the internal stability of the K = 2 solution. First, the model was refitted using 200 random seeds, retaining k-means++ initialization and n_init = 10 for each run. Cluster assignments were compared with the primary solution using the adjusted Rand index (ARI). Second, 200 random subsamples, each comprising 80% of the analytical sample (*n* = 7960), were drawn without replacement. Standardization was refitted independently within each subsample, followed by K-means clustering using the primary model settings. Agreement was calculated between the subsample assignments and the primary assignments for the same participants. The distributions of ARI values and the directions of the mean indicator profiles were examined. These analyses assessed internal membership stability rather than independent external validation.

To examine the influence of heterogeneous measurement characteristics, a separate sensitivity analysis was performed using Gower dissimilarity and the FasterPAM clustering algorithm in the full analytical sample (*n* = 9950). Self-rated health, sleep quality, and fatigue were treated as ordinal indicators, while the stair-climbing and 300-m walking items were treated as symmetric binary indicators. The remaining numerical indicators were scaled by their observed ranges, and equal weights were assigned to all ten indicators. Solutions for K = 2, 3, and 4 were examined using ten initializations for each K, with the solution showing the lowest total dissimilarity retained. Mean silhouette coefficients were calculated using the corresponding Gower dissimilarities. Agreement between the Gower-based K = 2 solution and the primary K-means solution was evaluated using ARI and the number of changed cluster assignments. Because the distance measures differed, silhouette coefficients from the Gower and Euclidean analyses were not treated as directly comparable measures of methodological superiority.

An additional indicator-selection sensitivity analysis was conducted by excluding the two binary mobility indicators (stair-climbing and 300-m walking ability) and refitting the K = 2 K-means model using the remaining eight standardized indicators. The primary K-means settings were retained, and agreement with the original cluster assignments was evaluated using ARI and the number and percentage of participants whose assignments changed. This analysis examined the extent to which the original partition depended on the inclusion of mobility-related indicators.

The primary clustering analysis was conducted using the unweighted analytical sample. Because the 2023 National Survey of Older Koreans employed a stratified cluster sampling design, a separate survey-weight sensitivity analysis was undertaken using the supplied weight variable (f_wgt1). The weights were normalized to a sample mean of one and incorporated into both variable standardization and the K-means objective. The resulting assignments were compared with the primary unweighted solution using ARI and assignment agreement. Weighted cluster proportions within the analytical sample were also calculated. This procedure assessed sensitivity to survey weighting but did not constitute full complex-survey inference, as stratification and primary sampling unit adjustments were not implemented. Accordingly, neither the unweighted nor weighted cluster proportions were interpreted as national prevalence estimates.

A further sensitivity analysis examined the definition of the weight-change indicator. The primary analysis used weight 1 year previously minus current weight, expressed in kilograms, with positive values indicating weight loss. The K = 2 model was refitted after replacing this indicator with proportional weight loss, calculated as (weight 1 year previously − current weight)/weight 1 year previously. The same 9950 participants and the other nine indicators were retained. Standardization and K-means clustering were repeated using the primary model settings, and agreement between the two solutions was assessed using ARI and the number of changed assignments. This additional analysis was performed using Python 3.12.14 and scikit-learn 1.8.0.

Profile characteristics were summarized separately according to their relationship to the clustering procedure. Age, sex, educational attainment, living arrangement, and activities of daily living (ADL) were examined as characteristics not used to derive the clusters. Continuous variables were presented as means and standard deviations and compared using independent-samples *t*-tests, while categorical variables were presented as frequencies and percentages and compared using Pearson’s chi-squared tests. Cohen’s d, calculated using the pooled standard deviation, and Cramér’s V were used to describe the magnitude of differences in continuous and categorical characteristics, respectively. These comparisons characterized the derived groups within the same analytical sample and were not regarded as independent or external validation. Selected clustering inputs and categorical characteristics derived from them were summarized separately for descriptive purposes.

For the ten cluster-defining indicators, standardized group means, standard deviations, and absolute Cohen’s d values were used to describe the observed magnitude of separation between the fitted clusters. The effect sizes were ranked descriptively, without conducting inferential significance tests on these indicators after cluster formation. For the two binary mobility indicators, Cohen’s d reflected differences in response distributions expressed on the standardized scale. Because all ten indicators contributed to cluster construction, their observed differences were not interpreted as independent predictors, determinants, measures of classification performance, or indicators of clinical importance.

Comparisons of characteristics not used for clustering were considered exploratory. Nominal two-sided *p*-values were reported without multiplicity adjustment, with *p* < 0.05 used as the conventional significance threshold. The interpretation emphasized effect sizes and descriptive differences rather than confirmatory statistical inference.

### 2.4. Ethical Considerations

This study was conducted in accordance with the Declaration of Helsinki and approved by the Institutional Review Board of Seokyeong University (SKUIRB-2025-02-004; approval date: 24 December 2025). Informed consent for participation in the 2023 NSOK and the use of survey data was obtained from the participants by the original survey investigators.

## 3. Results

### 3.1. Identification of Multidimensional Frailty-Related Profiles

The final analytical sample comprised 9950 participants, following the exclusion of 128 respondents from the original survey sample of 10,078. Compared with included participants, excluded respondents were older (81.97 ± 6.75 vs. 74.02 ± 6.75 years), had higher ADL scores (11.38 ± 4.31 vs. 7.18 ± 0.91), and were more likely to have no formal education (41.4% vs. 14.4%). The proportions of women were similar between the excluded and included groups (61.7% vs. 61.6%; Table S2). After standardization of the ten clustering variables, K-means solutions for K = 2–10 were compared using WCSS and mean silhouette coefficients.

**Figure 1:**
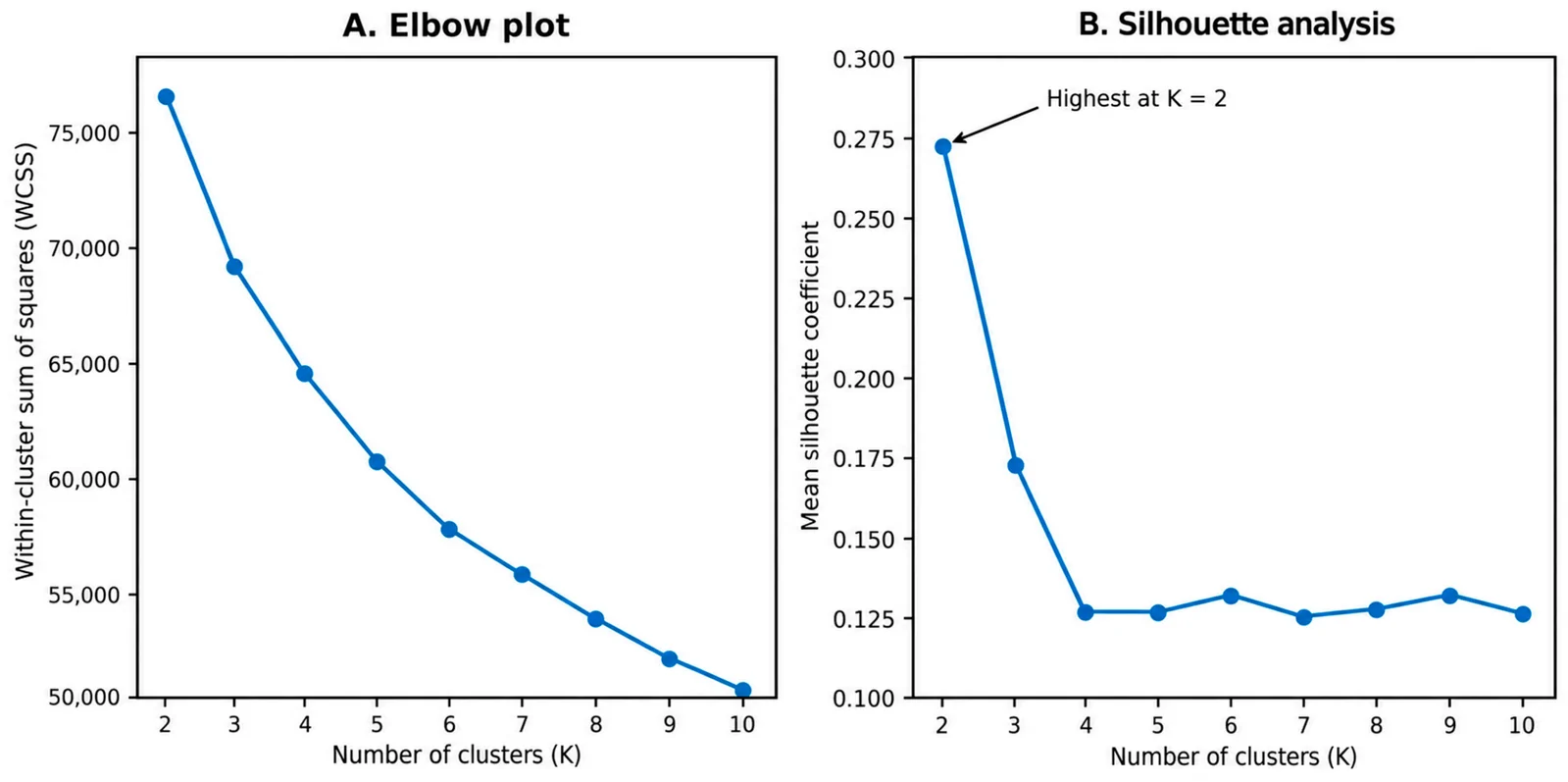
Determination of the optimal number of clusters. (**A**) Elbow plot based on the within-cluster sum of squares (WCSS). (**B**) Mean silhouette coefficients for K = 2–10.

The WCSS decreased from 76,803.735 at K = 2 to 69,059.269 at K = 3, representing a reduction of 7744.466 (10.1%), which was the largest proportional decrease across adjacent candidate solutions. WCSS then declined more gradually to 64,550.202 at K = 4, 60,658.145 at K = 5, 57,845.155 at K = 6, 55,819.089 at K = 7, 53,931.899 at K = 8, 52,073.098 at K = 9, and 50,454.382 at K = 10 (Figure 1A).

The mean silhouette coefficient was highest at K = 2 (0.273), decreased to 0.172 at K = 3, and remained between 0.126 and 0.132 across K = 4–10 (Figure 1B). Considering the silhouette coefficients, WCSS patterns, parsimony, and substantive interpretability, K = 2 was selected as the final solution. Nevertheless, the mean silhouette coefficient of 0.273 indicated modest separation between the derived groups. The proportion of total standardized variation attributable to between-cluster separation was 0.228 (BSS/TSS). Accordingly, the selected solution was interpreted as a parsimonious empirical partition of relative multidimensional vulnerability rather than evidence of clearly distinct underlying populations.

The K = 2 solution classified 6962 participants (70.0%) into Cluster 1 and 2988 (30.0%) into Cluster 2. Across the standardized centroids, Cluster 1 consistently showed more favorable values and Cluster 2 had less favorable values for the 10 indicators (Table 2). This pattern was interpreted as a gradient of multidimensional vulnerability rather than two qualitatively distinct clinical types. Therefore, Cluster 1 was labeled the lower-vulnerability profile, and Cluster 2 was labeled the multidimensional higher-vulnerability profile. The percentages represent the analytical sample and should not be interpreted as population prevalence estimates for older adults in Korea.

### 3.2. Characteristics of the Frailty-Related Profiles

The two clusters differed in characteristics that had not been used for cluster derivation (Table 3). Compared with the multidimensional higher-vulnerability profile, the lower-vulnerability profile had a lower mean age (72.36 ± 6.01 vs. 77.90 ± 6.80 years; *d* = 0.885), a lower proportion of women (57.5% vs. 71.1%; *V* = 0.128), and fewer participants living alone (29.4% vs. 46.1%; *V* = 0.161). Educational attainment also differed between the groups (*V* = 0.363). Mean ADL scores were 7.03 ± 0.31 and 7.52 ± 1.53, respectively (*d* = 0.560). All five comparisons had nominal *p*-values below 0.001. These comparisons were exploratory and did not constitute external validation.

Additional descriptive characteristics based on clustering inputs or their derived categories are presented separately in Table S1. The higher-vulnerability profile showed a greater chronic disease burden, higher depressive symptom scores, and greater proportions of participants reporting poor sleep, fatigue, poor self-rated health, or cognitive impairment. Because these characteristics were included in, or derived from variables used for, cluster construction, their distributions were interpreted as descriptive features of the fitted groups rather than independent confirmation of the clustering solution.

### 3.3. Indicators with the Largest Between-Profile Differences

Differences across the ten cluster-defining indicators were summarized using standardized group means, standard deviations, and absolute Cohen’s d values (Table 4). Because these indicators were used to derive the clusters, their observed differences describe separation within the fitted solution rather than independent associations with vulnerability. The effect sizes were therefore interpreted descriptively and were not considered evidence of predictive ability, clinical importance, or variable importance.

Among the cluster-defining indicators, the largest descriptive differences were observed for the ability to walk 300 m without assistance (|d| = 2.013) and climb 10 stairs (|d| = 2.001), followed by self-rated health (|d| = 1.582) and depressive symptoms (|d| = 1.418). Differences were smaller for chronic disease count (|d| = 1.077), sleep quality (|d| = 1.076), cognitive function (|d| = 0.998), fatigue (|d| = 0.763), weekly exercise frequency (|d| = 0.408), and 1-year weight change (kg; |d| = 0.270). These rankings reflect descriptive separation across the variables used to construct the clusters.

### 3.4. Stability and Sensitivity Analyses

Across 200 random-seed runs, the median ARI relative to the primary solution was 0.99794 (Q1–Q3, 0.99794–0.99835). In 200 repeated 80% subsamples, the median ARI was 0.99099 (Q1–Q3, 0.98615–0.99485). The direction of the ten mean vulnerability indicators was retained across these analyses (Table S3).

In the Gower/FasterPAM sensitivity analysis, the K = 2 solution yielded clusters of 7434 and 2516 participants. Agreement with the primary solution was ARI = 0.66236, with 886 participants (8.90%) assigned differently. Excluding both mobility indicators from the K-means model yielded clusters of 6228 and 3722 participants, with an ARI of 0.46129 and 1576 assignment changes (15.84%). These results indicate that the overall vulnerability gradient was retained, although individual cluster membership depended on the analytical specification (Table S4).

In the survey-weight sensitivity analysis, the weighted K-means solution yielded clusters containing 6895 and 3055 participants. Compared with the primary unweighted solution, 75 participants (0.75%) changed cluster assignment (ARI = 0.96935). The weighted proportions within the analytical sample were 70.41% and 29.59%, respectively. The direction of the ten mean vulnerability indicators was preserved (Table S4).

Replacing the original kilogram-based weight-change indicator with proportional weight loss yielded clusters of 6966 and 2984 participants. Eight participants (0.08%) changed assignment relative to the primary solution (ARI = 0.99670), and the mean silhouette coefficient was 0.27329 (Table S4).

## 4. Discussion

This cross-sectional analysis of 9950 participants identified two empirically derived groups characterized by relatively lower and higher multidimensional frailty-related vulnerability. Although the K = 2 solution had the highest mean silhouette coefficient among the candidate solutions, the value of 0.273 indicated modest separation. The higher-vulnerability group showed less favorable health, physical function, cognitive, and psychological characteristics. Among the indicators used to derive the clusters, the largest descriptive differences were observed for the ability to walk 300 m and climb 10 stairs without assistance, followed by self-rated health and depressive symptoms. These differences characterize the fitted clustering solution and should not be interpreted as independent evidence of clinical importance.

The two-cluster solution appeared to represent a partition along a multidimensional vulnerability continuum rather than two sharply distinct clinical frailty phenotypes. Across all ten indicators, the standardized group means followed a consistent direction from relatively favorable to less favorable health, functional, cognitive, and psychological characteristics. This pattern was broadly retained in the additional sensitivity analyses. Yet the limited separation between groups and shifts in membership under alternative specifications suggest that the partition is partly contingent on methodological choices. The resulting profiles describe co-occurring characteristics within this sample; they do not define diagnostic categories or successive stages of frailty.

The clearest contrasts involved walking 300 m and climbing 10 stairs without assistance, followed by self-rated health and depressive symptoms. As these indicators contributed to cluster formation, the contrasts reflect the fitted solution rather than independently established clinical relevance. In the sensitivity analysis excluding both mobility indicators, 1576 participants (15.84%) changed cluster assignment, with an adjusted Rand index of 0.46129 relative to the primary solution. Although the overall direction of the vulnerability gradient was retained, this finding indicates substantial dependence of individual cluster membership on the inclusion of mobility indicators. Furthermore, the survey did not directly assess the physiological components underlying mobility performance, limiting interpretation of the observed differences in terms of specific functional mechanisms.

The differences between the two profiles extended beyond mobility-related indicators. The higher-vulnerability profile was characterized by less favorable self-rated health, more depressive symptoms, a greater chronic disease burden, poorer sleep quality, and lower cognitive function. These characteristics were included in the clustering procedure, and their observed differences therefore describe the resulting multivariable partition rather than independently established associations. Previous reviews have reported relationships between frailty and depression, sleep disturbances, and cognitive impairment in older adults [9,10,11]. In the present analysis, the concurrent distribution of these indicators provides a descriptive account of how several vulnerability-related domains were concentrated within the same group. However, the cross-sectional design does not establish temporal ordering or causal relationships among these characteristics, nor does it demonstrate that the derived profiles provide additional clinical information beyond existing frailty assessment measures.

The observed vulnerability gradient can be considered alongside previous person-centered studies that have described heterogeneous frailty-related characteristics in older populations. Looman et al. identified six latent classes across multiple health domains [6], while Bohn et al. reported three early frailty profiles using factor analysis and latent profile analysis [7]. Studies involving older primary care and home-based populations have also identified different configurations of frailty-related characteristics [12,13]. In Korea, Han et al. identified four latent classes among 3824 older men using the 2023 NSOK and examined their associations with life satisfaction [8]. Their four-class solution and the present two-cluster solution are not directly comparable because the populations, input indicators, and clustering approaches differed. By including both women and men, the present study examined how health-related, functional, behavioral, cognitive, and psychological characteristics were distributed across the derived groups. The supplementary analyses further revealed an important distinction: the overall pattern remained similar, whereas individual assignments varied with the clustering method and indicator set. This combination of findings provides a more nuanced account of multidimensional vulnerability in the surveyed population, without establishing separate clinical frailty subtypes or demonstrating an advantage over existing summary measures.

The potential clinical relevance of these findings should be considered in relation to established frailty assessment approaches. Instruments such as K-FRAIL provide structured screening based on predefined frailty-related domains, whereas the present profiles were empirically derived from the joint distribution of ten survey indicators. Several of these indicators overlap conceptually with K-FRAIL domains, but this overlap does not establish agreement with the screening instrument or demonstrate additional clinical value. Previous work has emphasized the importance of frailty assessment and the use of complementary measurement approaches in older adult healthcare [14,15]. The present findings illustrate how these characteristics coincide within the analytical sample, but it remains unclear whether the resulting profiles offer meaningful information beyond existing screening tools. This question requires direct comparisons with established frailty measures and assessment of their incremental value in independently collected, longitudinal data before clinical application can be considered.

The study has several strengths. It drew on a large analytical sample from a nationwide survey and examined indicators spanning health status, physical function, health behavior, cognition, and psychological well-being. Candidate solutions from K = 2 to 10 were evaluated using WCSS and silhouette coefficients, while differences across the cluster-defining indicators were summarized on a common descriptive scale. The additional analyses provided a more detailed assessment of the selected solution. Repeated initializations and subsampling showed high internal membership stability, whereas the Gower-based analysis and exclusion of mobility indicators revealed appreciable differences in individual assignments. Examining these results together helps distinguish stability under a specified clustering procedure from sensitivity to methodological choices. The findings therefore provide a more qualified description of multidimensional vulnerability rather than evidence of a universally robust cluster structure.

Several limitations warrant consideration. The cross-sectional design precludes temporal or causal inference, and the derived profiles cannot be interpreted as stages or transitions in frailty progression. Although K = 2 provided the highest mean silhouette coefficient among the candidate solutions, the value of 0.273 indicated limited cluster separation [16]. High agreement across repeated initializations and subsamples should therefore not be taken as evidence of clearly distinct underlying populations. Moreover, the Gower-based analysis and the exclusion of mobility indicators produced appreciable changes in cluster membership, demonstrating sensitivity to the distance measure and indicator selection. Of the 10,078 survey respondents, 128 (1.3%) were excluded from the primary analysis. Compared with included participants, excluded respondents were older, had greater ADL dependence, and were more likely to have no formal education. These differences suggest potential underrepresentation of older adults with greater functional vulnerability, despite the relatively small proportion excluded. The primary clustering analysis was unweighted. Although the survey-weight sensitivity analysis showed high agreement with the primary assignments, it did not account for the full complex sampling design, including stratification and primary sampling units. Consequently, the observed cluster proportions should not be interpreted as national prevalence estimates. Finally, the profiles were derived and internally examined using the same survey dataset, without independent external validation.

Several indicators, including self-rated health, sleep quality, exercise frequency, and fatigue, relied on self-report and were therefore susceptible to reporting bias. The clustering variables were selected to reflect the core K-FRAIL domains while incorporating additional health-related, cognitive, and psychological measures available in the NSOK. However, the resulting framework did not directly assess social frailty, social isolation, the quality of social relationships, or social participation. Although living arrangement was examined as a profile characteristic, living alone cannot be regarded as a direct measure of social frailty. Previous research has highlighted the relevance of social frailty among community-dwelling older adults [17], while evidence from institutionalized older populations has also drawn attention to the relationships among frailty, multimorbidity, and physical health-related quality of life [18]. The present clustering model did not incorporate a direct measure of health-related quality of life. Consequently, the identified profiles capture selected dimensions of vulnerability rather than its full multidimensional scope. Different indicator sets could produce alternative profile structures, and the resulting groups should not be interpreted as comprehensive clinical frailty phenotypes.

Future investigations should establish whether similar vulnerability patterns emerge in independent populations and remain consistent across different analytical specifications. The present survey-weighted analysis showed high agreement with the primary solution, but its treatment of the sampling design was incomplete; changes in membership under alternative clustering methods also warrant further examination. Longitudinal studies could then determine whether group membership is associated with disability, falls, hospitalization, or mortality. Comparisons with established frailty instruments would help assess whether these empirical groupings provide additional information relevant to clinical assessment or preventive planning.

## 5. Conclusions

K-means clustering of 9950 participants from the 2023 National Survey of Older Koreans identified two groups representing relatively lower and higher multidimensional frailty-related vulnerability. Their consistent differences across health-related, functional, behavioral, cognitive, and psychological indicators were more compatible with a vulnerability gradient than with clearly distinct clinical frailty phenotypes. Although the primary solution showed high internal membership stability across repeated initializations and subsamples, individual assignments were sensitive to the clustering method and the inclusion of mobility indicators. The observed differences across cluster-defining variables describe the empirical partition and do not establish their independent clinical importance. These findings characterize the co-occurrence of selected vulnerability dimensions within the analytical sample. Independent replication, longitudinal outcome validation, and comparisons with established frailty assessment tools are required before the profiles’ additional clinical utility or suitability for individualized preventive strategies can be determined.

## Figures and Tables

**Table 1:** Classification of Variables Used for Clustering and Profile Characterization.

Category	Variables
Variables used for clustering (*n* = 10)	Self-rated health; chronic disease count; 1-year weight change (kg); sleep quality; weekly exercise frequency; fatigue item score; ability to climb 10 stairs without assistance; ability to walk 300 m without assistance; K-MMSE-2 score; SGDS-15 score
Non-clustering variables used for profile characterization	Age; sex; educational attainment; living arrangement; ADL score
Clustering inputs or derived descriptive characteristics	Chronic disease count; SGDS-15 score; categorized self-rated health; poor sleep; fatigue status; cognitive impairment status

Note. ADL, activities of daily living; K-MMSE-2, Korean Mini-Mental State Examination-2; SGDS-15, 15-item Short Geriatric Depression Scale. The third category includes original clustering inputs and categorical characteristics derived from clustering inputs. These variables were used for descriptive characterization and were not considered independent validation measures.

**Table 2:** Cluster Centroids of Multidimensional Frailty Indicators.

Domain	Indicator	Cluster 1 (*n* = 6962)	Cluster 2 (*n* = 2988)
Physical health	Self-rated health	−0.385	0.896
	Chronic disease count	−0.290	0.676
	1-year weight change (kg)	−0.081	0.188
	Sleep quality	−0.290	0.675
Health behavior	Weekly exercise frequency	0.121	−0.281
Frailty symptom	Fatigue item score	0.216	−0.504
Physical function	Ability to climb 10 stairs without assistance	0.443	−1.032
	Ability to walk 300 m without assistance	0.444	−1.035
Cognitive function	K-MMSE-2 score	0.273	−0.635
Psychological	SGDS-15 score	−0.357	0.832

Note. Values represent standardized cluster centroids (Z-scores). Higher values indicated a worse status for self-rated health, chronic disease count, 1-year weight-loss proportion, sleep quality, and SGDS-15; better status for weekly exercise frequency and K-MMSE-2; and less frequent fatigue for the fatigue item score. For the stair-climbing and 300-m walking items, the original coding was 1 = difficulty and 2 = no difficulty; therefore, higher standardized scores indicated better mobility. K-MMSE-2, Korean Mini-Mental State Examination-2; SGDS-15, 15-item Short Geriatric Depression Scale. Cluster 1 = lower-vulnerability profile; Cluster 2 = multidimensional higher-vulnerability profile.

**Table 3:** Non-Clustering Characteristics of the Two Frailty-Related Profiles.

Variables	Cluster 1 (*n* = 6962)	Cluster 2 (*n* = 2988)	*p*	Effect Size
Sociodemographic characteristics				
Age, years	72.36 ± 6.01	77.90 ± 6.80	<0.001	*d* = 0.885
Female, %	57.5	71.1	<0.001	*V* = 0.128
Educational level			<0.001	*V* = 0.363
No formal education	532 (7.6)	903 (30.2)		
Elementary school	1825 (26.2)	1094 (36.6)		
Middle school	1586 (22.8)	528 (17.7)		
High school	2465 (35.4)	395 (13.2)		
College or higher	554 (8.0)	68 (2.3)		
Living alone, %	29.4	46.1	<0.001	*V* = 0.161
Health and functional characteristics				
ADL score	7.03 ± 0.31	7.52 ± 1.53	<0.001	*d* = 0.560

Note. Values are presented as mean ± SD, *n* (%), or percentages, as appropriate. Between-cluster comparisons were exploratory. Continuous variables were compared using independent-samples *t*-tests, and categorical variables were compared using Pearson’s chi-squared tests. Effect sizes were expressed as Cohen’s d for continuous variables and Cramér’s V for categorical variables. All characteristics presented in this table were excluded from the clustering procedure. These comparisons should not be regarded as independent or external validation of the cluster solution. ADL, activities of daily living; SD, standard deviation.

**Table 4:** Frailty-Related Indicators Showing the Largest Between-Profile Differences.

Rank	Frailty-Related Indicator	Cluster 1 Mean ± SD	Cluster 2 Mean ± SD	Absolute Cohen’s d
1	Ability to walk 300 m without assistance	0.44 ± 0.40	−1.04 ± 1.19	2.013
2	Ability to climb 10 stairs without assistance	0.44 ± 0.63	−1.03 ± 0.95	2.001
3	Self-rated health	−0.38 ± 0.78	0.90 ± 0.87	1.582
4	Depressive symptoms	−0.36 ± 0.67	0.83 ± 1.13	1.418
5	Chronic disease count	−0.29 ± 0.80	0.68 ± 1.10	1.077
6	Sleep quality	−0.29 ± 0.85	0.68 ± 0.99	1.076
7	Cognitive function	0.27 ± 0.81	−0.63 ± 1.11	0.998
8	Fatigue item score	0.22 ± 0.89	−0.50 ± 1.06	0.763
9	Weekly exercise frequency	0.12 ± 1.00	−0.28 ± 0.94	0.408
10	1-year weight change (kg)	−0.08 ± 0.88	0.19 ± 1.21	0.270

Note. Values are standardized scores (Z-scores). The variables were ordered according to the absolute magnitude of Cohen’s d, with larger absolute values indicating greater between-profile differences. For the stair-climbing and 300-m walking items, higher scores indicated better mobility; for weekly exercise frequency and K-MMSE-2, higher scores indicated better status; for the fatigue item score, higher scores indicated less frequent fatigue; and for self-rated health, chronic disease count, 1-year weight change (kg), sleep quality, and SGDS-15, higher scores indicated worse status. Because these indicators were used to derive the clusters, the standardized differences and absolute effect sizes characterize separation within the fitted solution. They should not be interpreted as independent predictors, determinants, or measures of clinical importance. SD, standard deviation.

## Data Availability

The data analyzed in this study are available from the Health and Welfare Data Portal of the Korea Institute for Health and Social Affairs (KIHASA; https://data.kihasa.re.kr/) upon registration and completion of the required data-use application procedures.

## Supplementary Materials

The following supporting information can be downloaded at https://www.mdpi.com/article/doi/s1: Table S1: Clustering inputs and derived descriptive characteristics by profile; Table S2: Characteristics of included and excluded survey respondents; Table S3: Internal stability of the primary two-cluster K-means solution; Table S4: Sensitivity of the two-cluster solution to analytical specifications.

## Abbreviations

The following abbreviations are used in this manuscript:

NSOK	National Survey of Older Koreans
K-MMSE-2	Korean Mini-Mental State Examination-2
SGDS-15	15-item Short Geriatric Depression Scale
ADL	Activities of daily living
WCSS	Within-cluster sum of squared distances
SD	Standard deviation
ARI	Adjusted Rand Index
BSS	Between-Cluster Sum of Squares
TSS	Total Sum of Squares
