# Supplementary material for: Multidimensional Frailty-Related Vulnerability Profiles Among Community-Dwelling Older Adults: A Data-Driven Cluster Analysis of the 2023 National Survey of Older Koreans
Source: Healthcare (Basel). 2026 Oct 23;14(21):3585. doi: 10.3390/healthcare14213585 (PMC13763358; doi:10.3390/healthcare14213585)
Supplement: Supplementary file 1 [file healthcare-14-03585-s001.zip › healthcare-4605183-supplementary.pdf]

Supplementary Materials

Table S1. Clustering inputs and derived descriptive characteristics by profile.

Characteristic	Cluster 1 (n = 6,962)	Cluster 2 (n = 2,988)	Relationship to cluster derivation
Chronic disease count, mean ± SD	1.73 ± 1.27	3.27 ± 1.75	Direct clustering input
SGDS-15 score, mean ± SD	1.94 ± 2.18	5.79 ± 3.66	Direct clustering input
Self-rated health: good, n (%)	3,865 (55.5)	248 (8.3)	Categorized clustering input
Self-rated health: fair, n (%)	2,543 (36.5)	902 (30.2)	Categorized clustering input
Self-rated health: poor, n (%)	554 (8.0)	1,838 (61.5)	Categorized clustering input
Poor sleep, %	7.5	38.9	Dichotomized clustering input
Fatigue status, %	4.4	20.4	Dichotomized clustering input
Cognitive impairment status, %	18.0	40.1	Derived from K-MMSE-2

Note. Values were taken from the corresponding descriptive comparisons in the originally submitted manuscript. Chronic disease count and SGDS-15 were direct clustering inputs; the remaining listed characteristics were categorical representations or derivatives of clustering inputs. These descriptive comparisons do not constitute independent validation. Self-rated health was grouped as good, fair, or poor. Abbreviations: K-MMSE-2, Korean Mini-Mental State Examination-2; SGDS-15, 15-item Short Geriatric Depression Scale; SD, standard deviation.

Supplementary Materials

Table S2. Characteristics of included and excluded survey respondents.

Characteristic	Included (n = 9,950)	Excluded (n = 128)	Standardized mean difference
Age, years, mean ± SD	74.02 ± 6.75	81.97 ± 6.75	1.177
ADL score, mean ± SD	7.18 ± 0.91	11.38 ± 4.31	1.346
No formal education, %	14.4	41.4	Not calculated
Women, %	61.6	61.7	Not calculated

Note. The original survey included 10,078 respondents. The primary analytical sample comprised 9,950 participants; 128 were excluded. Descriptive comparisons use available demographic and functional characteristics. Standardized mean differences are shown for continuous variables; no inferential p-values are presented. The differences do not establish the cause of exclusion. Abbreviations: ADL, activities of daily living; SD, standard deviation.

Supplementary Materials

Table S3. Internal stability of the primary two-cluster K-means solution.

Resampling procedure	Repetitions	Subsample size	Median ARI	Q1–Q3 ARI	Minimum–maximum ARI	Direction of ten mean indicator profiles
Random-seed refits	200	9,950	0.99794	0.99794–0.99835	0.99753–1.00000	Preserved in all runs
80% without-replacement subsamples	200	7,960	0.99099	0.98615–0.99485	0.95548–0.99897	Preserved in all runs

Note. ARI values quantify assignment agreement with the primary K-means solution. For each 80% subsample, standardization was refitted and agreement was calculated for the same participants. Profile direction was preserved for all ten mean indicators across the repeated analyses. These procedures assess internal stability, not external validation. Abbreviations: ARI, adjusted Rand index; Q1–Q3, first to third quartiles.

Supplementary Materials

Table S4. Sensitivity of the two-cluster solution to analytical specifications.

Specification	Cluster 1, n	Cluster 2, n	ARI vs. primary	Changed membership, n (%)	Additional result
Primary Euclidean K-means, 10 indicators	6,962	2,988	1.00000	0 (0.00%)	Silhouette = 0.27252
Gower dissimilarity + FasterPAM, 10 indicators	7,434	2,516	0.66236	886 (8.90%)	Gower silhouette (K = 2) = 0.48784
Euclidean K-means, 8 indicators (both mobility items excluded)	6,228	3,722	0.46129	1,576 (15.84%)	Overall vulnerability direction preserved
Survey-weighted standardization and K-means, 10 indicators	6,895	3,055	0.96935	75 (0.75%)	Weighted sample shares: 70.41% / 29.59%
Proportional-weight-loss definition in place of kg weight change	6,966	2,984	0.99670	8 (0.08%)	Silhouette = 0.27329

Note. All analyses involved the same 9,950 participants. Changes in membership were calculated relative to the primary unweighted K-means solution. For the primary analysis, weight change was defined as weight one year earlier minus current weight (kg); proportional weight loss was tested only as a sensitivity analysis. The Gower-based silhouette is not directly comparable to the Euclidean silhouette. Survey weights were applied to standardization and the K-means objective, without full complex-survey inference. Abbreviations: ARI, adjusted Rand index; FasterPAM, fast partitioning around medoids.
